# Effect of *Echium* oil compared with marine oils on lipid profile and inhibition of hepatic steatosis in LDLr knockout mice

**DOI:** 10.1186/1476-511X-12-38

**Published:** 2013-03-19

**Authors:** Patrícia Borges Botelho, Karina da Rocha Mariano, Marcelo Macedo Rogero, Inar Alves de Castro

**Affiliations:** 1 LADAF. NAPAN. Department of Food and Experimental Nutrition, Faculty of Pharmaceutical Sciences, University of São Paulo, Av. Lineu Prestes, 580, São Paulo B14 - 05508-900, Brazil; 2 Department of Nutrition, Faculty of Public Health, University of São Paulo, São Paulo, Brazil

**Keywords:** Atherosclerosis, Inflammation, Echium, Stearidonic, Omega 3, Steatosis

## Abstract

**Background:**

In an effort to identify new alternatives for long-chain n-3 polyunsaturated fatty acids (LC n-3 PUFA) supplementation, the effect of three sources of omega 3 fatty acids (algae, fish and *Echium* oils) on lipid profile and inflammation biomarkers was evaluated in LDL receptor knockout mice.

**Methods:**

The animals received a high fat diet and were supplemented by gavage with an emulsion containing water (CON), docosahexaenoic acid (DHA, 42.89%) from algae oil (ALG), eicosapentaenoic acid (EPA, 19.97%) plus DHA (11.51%) from fish oil (FIS), and alpha-linolenic acid (ALA, 26.75%) plus stearidonic acid (SDA, 11.13%) from *Echium* oil (ECH) for 4 weeks.

**Results:**

Animals supplemented with *Echium* oil presented lower cholesterol total and triacylglycerol concentrations than control group (CON) and lower VLDL than all of the other groups, constituting the best lipoprotein profile observed in our study. Moreover, the *Echium* oil attenuated the hepatic steatosis caused by the high fat diet. However, in contrast to the marine oils, *Echium* oil did not affect the levels of transcription factors involved in lipid metabolism, such as Peroxisome Proliferator Activated Receptor α (PPAR α) and Liver X Receptor α (LXR α), suggesting that it exerts its beneficial effects by a mechanism other than those observed to EPA and DHA. *Echium* oil also reduced N-6/N-3 FA ratio in hepatic tissue, which can have been responsible for the attenuation of steatosis hepatic observed in ECH group. None of the supplemented oils reduced the inflammation biomarkers.

**Conclusion:**

Our results suggest that *Echium* oil represents an alternative as natural ingredient to be applied in functional foods to reduce cardiovascular disease risk factors.

## Background

The increased intake of omega-6 fatty acids during the 20th century as a result of an elevation in vegetal oil consumption (of more than 1,000-fold) contributed to a decline in the tissue concentration of long-chain n-3 polyunsaturated fatty acids (LC n-3 PUFA) [1,2], which might be associated with the increased incidence of inflammatory disorders, such as atherosclerosis [3]. The development of atherosclerotic plaques is associated with several clinical cardiovascular events. Considering the health effects of LC n-3 PUFA toward the reduction of cardiovascular disease (CVD) risk [4,5], many industries have added eicosapentaenoic acid (EPA) and docosahexaenoic acid (DHA) from marine oils to food formulations or supplements, aiming to explore this health claim. In 2004, the Food and Drug Administration (FDA) qualified the health claim of products containing EPA and DHA [6]. A similar recommendation was also provided by the American Heart Association (AHA), who suggested consumption of 1 g/day of EPA + DHA for patients with CVD and 2–4 g/day for patients with hypertriglyceridaemia [7].

The cardioprotective effects of LC N-3 PUFA appear to be due to a synergism between multiple mechanisms including triacylglycerol (TG) lowering, improving membrane fluidity, anti-inflammatory, antiarrhythmic and antithrombotic effects [5]. The scientific evidence concerning the beneficial effects of the LC N-3 PUFA on lipid profile and inflammation were obtained from several studies using animal and human models. However, these effects and the mechanisms by which they occur are restricted to the action of EPA and DHA [8]. Other non-marine sources of omega 3 fatty acids (N-3 FA), such as alpha- linolenic acid (ALA) or stearidonic acid (SDA), can be converted *in vivo* to EPA and DHA by the desaturase and elongase enzymes in a tissue-dependent manner, the liver being the major site of this conversion [9]. It has been reported that the conversion rate of ALA is low (5-10% for EPA and < 1% for DHA), which diminishes the efficacy of these alternative sources in the reduction of cardiovascular risk [10-12]. However, due to dietary preferences, safety, sustainability, cost and oxidative stability aspects, other non-marine oils alternatives must be evaluated [3,9-11,13,14]. It has been suggested that the low rate by which ALA is converted to EPA is a result of the limited activity of Δ6-desaturase when linoleic acid (LNA) is also present [15]. However SDA, a precursor of EPA that is found in plants, such as *Echium* (*Echium plantagineum*), black currant seed and other genetically modified seeds, does not need Δ6-desaturase activity to be converted into EPA [3,10]. In an effort to identify new alternatives for LC N-3 PUFA supplementation, the objective of this study was to compare the effects of three sources of N-3 FA (algae, fish and *Echium* oil) on lipid composition and some inflammatory biomarkers using LDL receptor deficient mice (LDLr knockout mice) as model.

## Methods

### Oils and reagents

The N-3 FA used in this study were commercial products: the algae oil containing 40% DHA (DHASCO) was obtained from Martek Biosciences® (Winchester, KY, USA), the fish oil (EPA1T1600 MEG-3™) containing EPA (20%) + DHA (12%) was obtained from Ocean Nutrition® (Dartmouth, NS, Canada) and the *Echium* oil containing 11.5% SDA (AW39144ECH) was obtained from Oil Seed Extraction® (Ashburton, New Zealand). All reagents were purchased from Sigma Chemical Co. (St. Louis, MO, USA), Merck (Darmstadt, Germany), Calbiochem Technology Inc. (Boston, MA, USA) and GE Healthcare (Little Chalfont, Bucks, UK). The aqueous solutions were prepared with ultra-pure Milli-Q water (Millipore Ind. Com. Ltd., SP, Brazil), and the organic solvents were of HPLC grade. Fatty acids profile of the oils used in this study was analyzed by gas chromatography and is shown in Table 1.

**Table 1:** Fatty acids composition of the edible oils applied in this study

**Fatty acids (g/100 g total FA)**	**Fish oil**	**Algae oil**	***Echium *oil**
C10:0 – capric	-	1.12	-
C12:0 – lauric	-	4.92	-
C14:0 – miristic	7.93	10.19	-
C16:0 – palmitic	17.05	6.97	5.15
C18:0 – stearic	3.05	0.92	2.57
C16:1 – palmitoleic	9.76	2.82	-
C18:1 - oleic (n-9)	13.20	27.48	12.77
C18:2 – (LNA) linoleic (n-6)	1.19	1.17	27.52
C18:3 – (ALA) α-linolenic (n-3)	0.68	-	26.75
C18:3 – (GLA) γ- linolenic (n-6)	-	-	13.11
C18:4 – (SDA) stearidonic (n-3)	3.28	-	11.13
C20:4- (ARA) arachidonic (n-6)	1.82	-	-
C20:5 – (EPA) eicosapenatenoic (n-3)	19.97	-	-
C22:5 – (DPA) docosapentaenoic (n-3)	2.20	0.38	-
C22:6 – (DHA) docosaexaenoic (n-3)	11.51	42.89	-
Σ saturated FA	28.03	24.12	7.72
Σ monounsaturated FA	22.96	30.30	12.77
Σ polyunsaturated FA	40.65	44.44	78.51
N-6 FA	3.01	1.17	40.63
N-3 FA	37.64	43.27	37.88
N-6/N-3 FA ratio	0.08	0.03	1.07

### Animals and diets

Forty male homozygous LDL receptor-deficient mice (LDLr Knockout mice, C57BL/6) weighing 25–29 g (4.0-4.5 months of age) were purchased from the Faculty of Pharmaceutical Sciences (São Paulo, Brazil). The mice were housed in plastic cages (5 animals/cage) at constant temperature (22 ± 2°C) and relative humidity (55 ± 10%), with a 12-h light–dark cycle. Food and water were available *ad libitum*, and animals were divided into four groups. All groups were fed with a high fat diet for 4 weeks (Table 2), and were supplemented with an oil-in-water emulsion (190 – 240 μL/d) per mouse containing fish oil (FIS), algae oil (ALG), *Echium* oil (ECH) or water (CON) by gavage. Amount of EPA, DHA, SDA and ALA ingested from added oil per day is presented in Table 3. We opted to compare the effect of the same amount of EPA+DHA in all groups. To achieve this objective the rate of conversion from SDA to EPA (4:1) proposed by Whelan [16] was adopted in our study. By this way, the EPA+DHA dosage applied was 0.7, 0.8 and 0.7 mg/day using Fish, Alagae and *Echium* oil respectively (Table 3). The emulsions were prepared weekly by mixing the respective oil with water using a high-pressure homogenizer (Homolab mod A-10, Alitec, São Paulo, Brazil). The emulsions were prepared in less than 2 minutes, and the temperature was kept below 40°C, during this short time. After, the emulsions were transferred to 2 mL eppendorf tubes, immediately immersed in nitrogen and kept at -80°C until the time of gavage. All this procedure was repeated twice a week. Emulsion characteristics are presented in Table 3. Diet consumption was measured daily and animals were weighed individually twice a week. After 4 weeks, the mice were fasted for 12 h, and anaesthetised with a mixture containing xylazine 2% (Sespo Ind. e Com. Ltda., Paulínia, Brazil), ketamine (Syntec do Brasil Ltda., Cotia, Brazil) and acepromazine (Vetnil Ind. e Com. de Prod. Veterinários Ltda., Louveira, Brazil). Blood samples collected from the brachial plexus were immediately centrifuged (1,600 × g for 15 min at 4°C), frozen under liquid nitrogen and stored (-80°C) for further analysis. The liver was excised, dried with lint and weighed. Small pieces of the larger lobe were frozen for Western blotting and further analysis, and a piece of the smaller lobe was immersed in 10% buffered formalin solution for histopathological examination. Subsequently, the animals were perfused with a cold NaCl solution (0.9%, 240.0 mL) via their left ventricle to remove the excess of blood. The animal protocol was approved by the Ethics Committee for Animal Studies of the Faculty of Pharmaceutical Sciences (São Paulo, Brazil).

**Table 2:** Composition and major fatty acids profile of the high fat diet

**Composition**^**2**^	**High fat diet**^**1**^
Moisture (g/100 g)	7.22 ± 0.05
Ashes (g/100 g)	2.94 ± 0.10
Protein (g/100 g)	17.99 ± 0.49
Lipids (g/100 g)	30.72 ± 0.57
Carbohydrate (g/100 g)	41.13 ± 0.33
Energy (Kcal/100 g)	512.96 ± 3.32
Fatty acids (g/100 g total FA)	
C10:0 – capric	0.81 ± 0.00
C12:0 - lauric	1.03 ± 0.17
C14:0 - miristic	1.68 ± 0.01
C16:0 - palmitic	26.20 ± 0.62
C18:0 - stearic	17.30 ± 0.05
C16:1 – palmitoleic	0.96 ± 0.07
C18:1 n-9 - oleic	32.73 ± 0.12
C18:2 – (LNA) linoleic (n-6)	11.94 ± 0.97
C18:3 – (ALA) α-linolenic (n-3)	0.60 ± 0.06
C18:3 – (GLA) γ- linolenic (n-6)	0.25 ± 0.06
C18:4 – (SDA) stearidonic (n-3)	-
C20:0 - arachidic	0.27 ± 0.01
C20:1 - eicosaenoic (n-9)	0.59 ± 0.05
C20:2- eicosadienoic (n-6)	0.26 ± 0.02
C20:4- (ARA) arachidonic (n-6)	-
C20:5 – (EPA) eicosapenatenoic (n-3)	-
C22:5 – (DPA) docosapentaenoic (n-3)	-
C22:6 – (DHA) docosaexaenoic (n-3)	-
NI	5.15

^1 ^Based on Safwat et al. [30].

^2 ^Diet was composed by 187.0 g casein, 133.5 g sucrose, 300.0 g lard, 193.5 g corn starch, 67.0 g cellulose, 53.5 g soybean oil, 46.5 g minerals, 2.4 g L-cystine, 3.3 g choline bitartrate and 13.35 g vitamin mix/ kg diet.

NI, not identified.

**Table 3:** Characteristics of the emulsions prepared with three N-3 FA sources

	**Emulsions**		
**Characteristics**^**1**^	**Fish**	**Algae**	***Echium***
Emulsion volume (μL/d)	240	190	270
Oil in the emulsion (%)	1	1	10
Oil supplementation (μL/d)	2.4	1.9	27.0
Oil supplementation (mg/d)	2.2	1.8	25.1
C20:5 – (EPA) eicosapenatenoic (n-3) (mg/d)	0.44	-	-
C22:6 – (DHA) docosaexaenoic (n-3) (mg/d)	0.25	0.77	-
C18:4 – (SDA) stearidonic (n-3) (mg/d)	0.07	-	2.79^2^
C18:3 – (ALA) linolenic (n-3) (mg/d)	0.01	-	6.71
C22:5 – (DPA) docosapentaenoic (n-3) (mg/d)	0.05	0.01	-
N-6 FA from gavage (mg/d)	0.07	0.02	10.20
N-3 FA from gavage (mg/d)	0.82	0.78	9.50^1^
N-6 FA from diet (mg/d)	80.07	80.02	80.00
N-3 FA from diet (mg/d)	4.00	4.00	4.00
Total N-6 FA (mg/d)	80.07	80.02	90.20
Total N-3 FA (mg/d)	4.82	4.78	13.50
N-6/N-3 FA ratio	16.6	16.7	6.7

^1 ^Supplementation is expressed as mg/d/animal.

^2 ^Assumed no conversion from ALA to EPA and equivalence from SDA to EPA of 25%, according to Whelan [16], resulting in about 0.70 mg/d.

### Measurements

Fatty acids were isolated from the liver and diets using the extraction methodology proposed by the Association of Official Analytical Chemists (method 996.06) [17]. The fatty acid methyl esters (FAME) were suspended in hexane and analyzed by a gas chromatograph (GC17A Shimadzu Class CG, Kyoto, Japan) equipped with a 30 m × 0.25 mm (i.d.), 0.25 μm film thickness fused silica capillary column (Supelcowax, Bellefont, PA, USA) and a flame ionization detector. Helium was used as a carrier gas, and the fatty acids were separated using a 10°C/min gradient from 80 to 150°C and then a 6°C/min gradient from 150°C to 230°C. Standard mixtures with 37 FAME and PUFA 3 methyl esters from Menhaden oil (Sigma Chemical, St. Louis, MO, USA) was used to identify the peaks. The results were expressed as percentage of the total fatty acids present.

The serum lipoprotein concentrations, including total cholesterol, high density lipoprotein (HDL) and TG, were quantified using an enzymatic colorimetric test from Labtest (Lagoa Santa, MG, Brazil). The low density lipoprotein levels (LDL) and VLDL were estimated using the Friedewald formula [18]. The inflammation biomarkers (C-reactive protein (CRP), interleukin–6 (IL-6) vascular cell-adhesion molecule-1 (VCAM), Inter-Cellular Adhesion Molecule (ICAM) and adiponectin) were analysed in serum samples using Multiplex commercial Kits (Millipore, St. Charles, MO, USA).

### Liver histology

The representative liver fragments were fixed in a 10% buffered formalin solution for approximately 48 hours. Then, the fragments were fixed in paraffin. The material was submitted to microtomy with a cut of 5 μm and stained with hematoxylin and eosin for histopathological evaluation [19].

### Western blotting analysis of hepatic peroxisome proliferator activated receptor α (PPARα) and liver X receptor α (LXRα)

The total nuclear protein was extracted from the frozen liver tissue samples using the specific commercial Kit NEPER (GE Healthcare, Little Chalfont, Bucks, UK). Subsequently, 15 μg of the proteins present in the supernatant was separated on a 10% SDS-PAGE gel and transferred to Amersham™ Hybond™-ECL™ nitrocellulose membranes (GE Healthcare UK Ltd.-Amershan Place, Little Chalfont, Buckinghamshire, UK) using a humid system composed of buffer containing 25 mM Tris base, 192.0 mM glycine, 0.02% SDS, and 10% methanol (GE Healthcare, Little Chalfont, Bucks, UK). The membranes were then blocked with 5% ECL advance blocking agent in TBST (Tris-buffered saline and Tween 20) for 1.5 h to prevent the occurrence of nonspecific binding, and they were incubated with primary antibodies (rabbit polyclonal to LXRα – ab 82774 and rabbit polyclonal to PPARα – ab 8934, ABCAM plc., Cambridge, MA, USA) diluted in a ratio of 1:345 and 1:500, respectively. After this step, the membranes were incubated for 1 h at 4°C with a secondary antibody conjugated to horseradish peroxidase (GE Healthcare, Little Chalfont, Bucks, UK). Following three washes in TBST, the immunoreactive bands were visualised using the ECL advance detection system (Amersham Biosciences, Pittsburgh, PA, USA). To standardise the immunoblots, a digital detection system was applied (IMAGE QUANTTM 400 version 1.0.0, Amersham Biosciences, Pittsburgh, PA, USA) using proliferating cell nuclear antigen – PCNA (ABCAM plc., Cambridge, MA, USA) as standard. The densitometry was quantified using the Discovery series™ Quantity one® Analysis Software version 4.6.3 (Bio-Rad Laboratories Inc., Hercules, CA, USA).

### Statistical analysis

The effect of each N-3 FA source on biomarkers was compared by one-way ANOVA and Tukey HSD test. Equivalent non-parametric ANOVA was applied when there was no homogeneity of variances (Hartley test). A probability value of 0.05 was adopted to reject the null hypothesis. All calculations and graphs were performed using the software Statistica v.9 (Statsoft Inc., Tulsa, USA).

## Results

**Figure 1:**
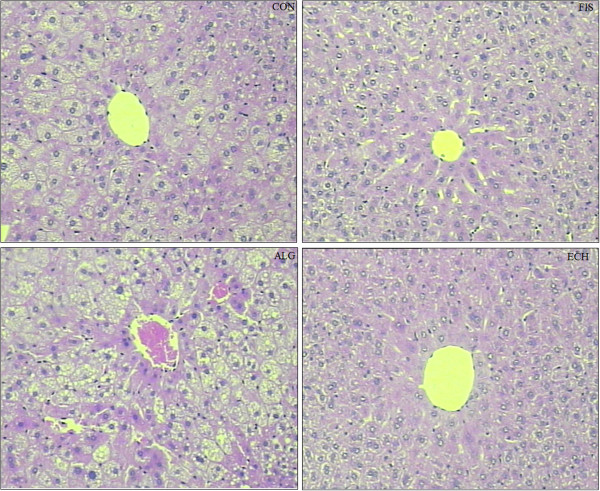
**Representative photomicrographs of liver sections: (CON) - fatty infiltration around the portal space; (FIS) and (ECH) - antisteatogenic effect exhibiting well-defined cells and low-fat vacuoles in the cytoplasm, and (ALG): hepatocytes presenting fatty infiltration around the portal space. **Original magnification 10X.

**Figure 2:**
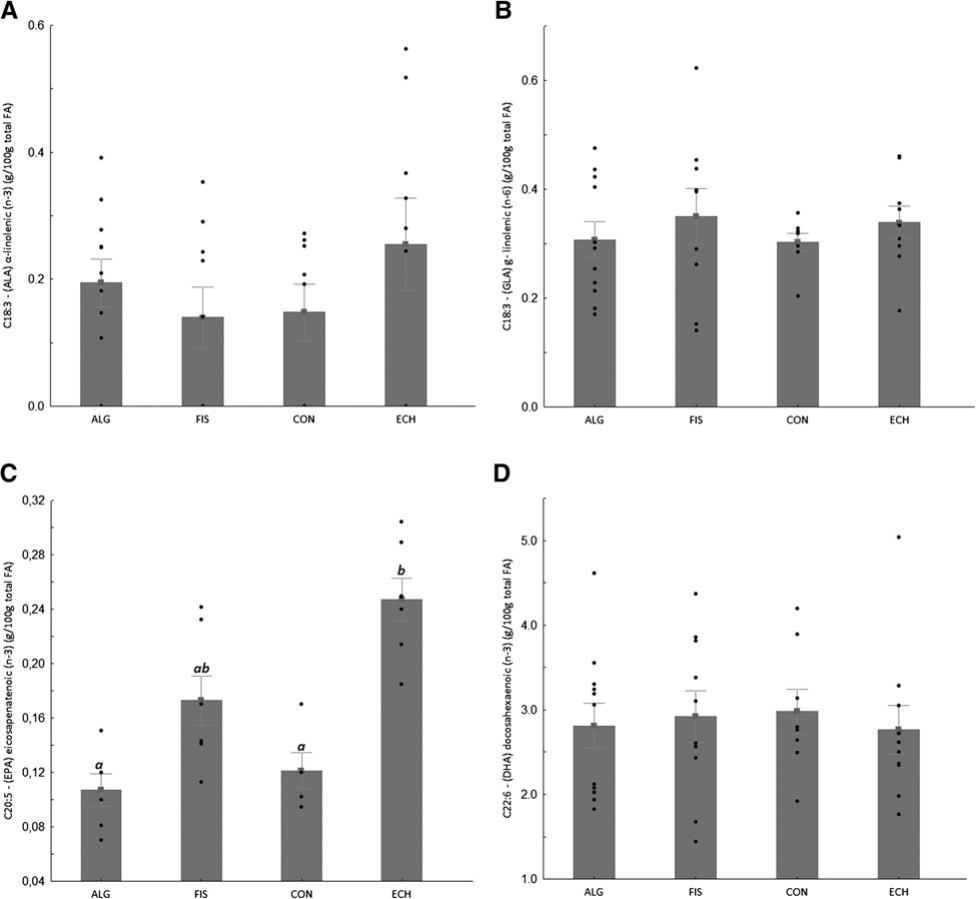
**Fatty acids content in the animal liver after the trial: (A) C18:3 – (ALA) α-linolenic (n-3), (B) C18:3 – (GLA) γ- linolenic (n-6); (C) C20:5 – (EPA) eicosapenatenoic (n-3) and (D) C22:6 – (DHA) docosaexaenoic (n-3) - water (CON), fish oil (FIS), algae oil (ALG) and Echium oil (ECH). **Bars followed by the same upperscrit letter do not differ (P<0.05). Data are mean±SE (raw data).

**Figure 3:**
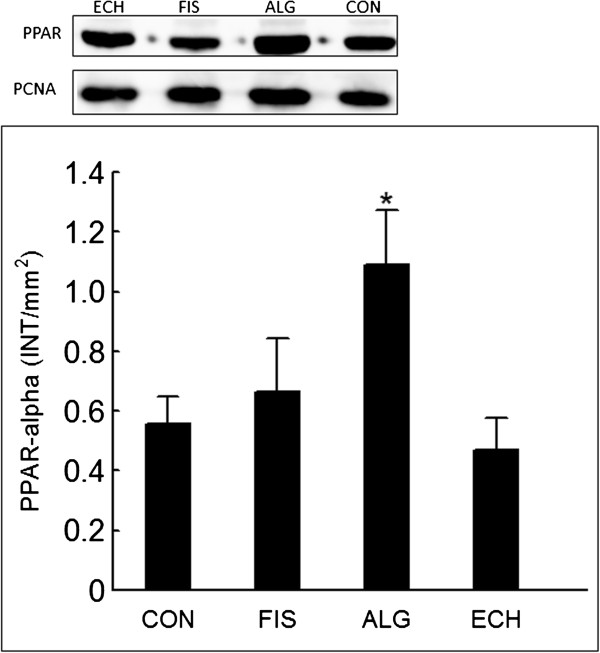
Changes in PPARα expression after 4 weeks of supplementation - water (CON), fish oil (FIS), algae oil (ALG) and *Echium *oil (ECH). *P<0.05.

**Figure 4:**
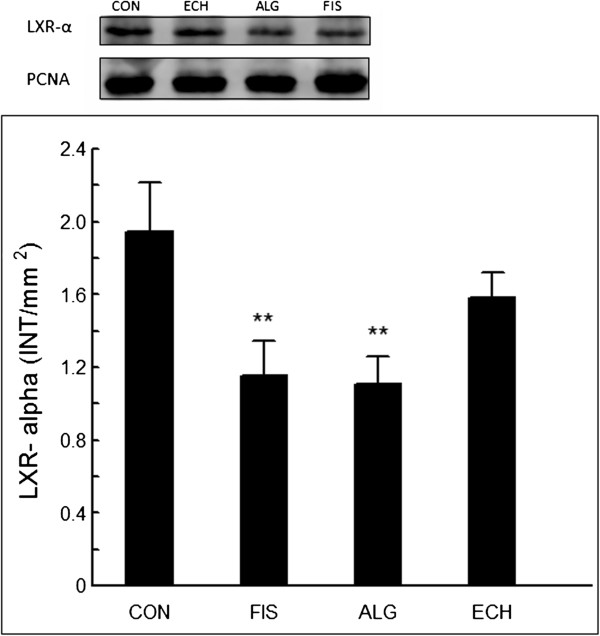
**Changes in LXRα expression after 4 weeks of supplementation- water (CON), fish oil (FIS), algae oil (ALG) and *Echium *oil (ECH).** **P<0.01.

All groups showed the same weight gain and diet consumption (Table 4). Animals supplemented with *Echium* oil presented lower total cholesterol and triacylglycerol concentrations than Control, and lower VLDL than all of the other groups, constituting the best lipoprotein profile observed in this study. None of the N-3 FA sources altered any of the inflammation biomarkers (Table 4). The effect of N-3 FA associated with high fat diet on the histological evaluation of the liver tissue is presented in Figure 1. Hepatic steatosis can be observed in the CON group with fatty infiltration around the portal space. Fish and *Echium* oils attenuated hepatic steatosis, whereas algae oil did not promote any protection against the hepatic steatosis induced by the high fat diet. The N-3 FA must be present in liver to exert their effects on lipid metabolism. Of the main N-3 FA applied in our study (ALA, SDA, EPA and DHA) (Figure 2), only EPA was observed in a higher concentration in the liver homogenate of animals supplemented with fish and *Echium* oils (Table 5). Moreover, a lower omega 6/ omega 3 ratio (N-6/N-3 FA ratio) (p<0.001) was observed in liver of animals supplemented with *Echium* oil (Table 5). In order to better investigate the action of N-3 FA on the hepatic steatosis, two transcription factors involved in lipid metabolism were evaluated. Figures 3 and 4 present the influence of the N-3 FA supplementation on the hepatic transcription factors LXRα and PPARα, which are associated with fatty acids synthesis and oxidation, respectively. Animals supplemented with algae oil experienced a significant increase of PPARα expression when compared to all other groups (Figure 3). A decrease in LXRα expression was observed in the groups supplemented with fish and algae oils (Figure 4), whereas *Echium* oil did not alter any of these two transcription factors.

**Table 4:** Body weight, diet consumption, plasma lipid profile and inflammatory biomarkers observed in the supplemented groups

	**CON**	**FIS**	**ALG**	**ECH**	***P***^***1***^
Initial weight (g)	26.9 ± 1.3	26.8 ± 0.6	27.3 ± 0.1	27.8 ± 0.8	0.670
Final weight (g)	28.9 ± 2.0	28.3 ± 0.6	29.8 ± 1.4	28.8 ± 0.5	0.731
Weight gain (g)	2.0 ± 0.7	1.5 ± 0.0	2.5 ± 1.3	1.1 ± 1.3	0.542
Diet consumption (g/d/mouse)	2.3 ± 0.2	2.3 ± 0.1	2.3 ± 0.1	2.4 ± 0.3	0.886
Cholesterol (mg/100 mL)	371.7 ± 37.4^a^	349.8 ± 37.2^ab^	350.2 ± 56.3^ab^	312.9 ± 40.2^b^	0.040
LDL (mg/100 mL)	244.3 ± 32.2	240.4 ± 34.2	227.6 ± 50.4	199.3 ± 31.4	0.054
HDL (mg/100 mL)	95.9 ± 8.8	82.7 ± 13.7	95.0 ± 8.4	92.0 ± 13.7	0.050
VLDL (mg/100 mL)	29.7 ± 4.6^a^	26.7 ± 5.1^a^	27.6 ± 9.7 ^a^	21.6 ± 3.5^b^	0.036
Triacylglicerol (mg/100 mL)	149.0 ± 23.6^a^	133.6 ± 25.4^ab^	115.8 ± 34.2^b^	109.1 ± 17.5 ^b^	0.008
CRP (ng/mL)	121.6 ± 5.5	118.9 ± 6.7	118.0 ± 9.6	124.6 ± 8.6	0.232
IL-6 (pg/mL)	11.4 ± 2.3	10.6 ± 2.0	10.1 ± 1.9	11.2 ± 2.0	0.457
VCAM (pg/mL)	1.7 ± 0.4	1.7 ± 0.3	1.6 ± 0.5	1.3 ± 0.2	0.059
ICAM (ng/mL)	39.3 ± 6.7	36.9 ± 3.8	39.2 ± 4.4	35.4 ± 3.4	0.188
ADIPONECTIN (pg/mL)	13.9 ± 8.3	14.4 ± 6.0	11.5 ± 3.7	14.3 ± 9.3	0.805

^1 ^Probability value obtained by ANOVA. Values followed by the same letter are not significantly different at the 0.05; a,b: p<0.05(n = 10/group).

**Table 5:** Major fatty acids composition in liver homogenate (g/100 g Total FA) observed in the supplemented groups

**Fatty acids**	**CON**	**FIS**	**ALG**	**ECH**	**P**^**1**^
C14:0 – myristic acid	0.47 ± 0.08	0.46 ± 0.06	0.50 ± 0.08	0.41 ± 0.08	0.079
C16:0 – palmitic acid	26.37 ± 1.96	28.73 ± 4.15	27.60 ± 3.20	29.99 ± 3.91	0.163
C18:0 – stearic acid	12.11 ± 3.85	13.43 ± 4.98	10.50 ± 2.74	15.61 ± 6.43	0.107
C18:1- oleic acid	27.09 ± 6.25	26.4 ± 6.33	29.5 ± 4.87	22.15 ± 7.34	0.074
C18:2 – (LNA) linoleic (n-6)	12.87 ± 3.22	11.40 ± 4.82	12. 53 ±5.81	10.07 ± 4.64	0.584
C18:3 – (ALA) α-linolenic (n-3)	0.15 ± 0.13	0.14 ± 0.14	0.19 ± 0.12	0.26 ± 0.22	0.744
C18:3 – (GLA) γ-linolenic (n-6)	0.30 ± 0.05	0.35 ± 0.15	0.31 ± 0.11	0.34 ± 0.09	0.396
C20:4- (ARA) arachidonic (n-6)	6.69 ± 1.85	5.31 ± 1.46	4.80 ± 1.26	4.55 ± 1.22	0.060
C20:5 – (EPA) eicosapenatenoic (n-3)	0.12 ± 0.04^a^	0.17 ± 0.06^ab^	0.1 1± 0.05^a^	0.25 ± 0.04^b^	0.004
C22:6 –(DHA) docosahexaenoic (n-3)	2.98 ± 0.75	2.92 ± 0.96	2.8 1± 0.88	2.76 ± 0.92	0.951
N-6 FA	19. 86 ± 3.56^b^	17.06 ± 5.78^ab^	17.64 ± 6.61^ab^	14.97 ± 5.81^a^	0.017
N-3 FA	3.25 ± 0.70	3.23 ± 0.98	3.12 ± 0.95	3.27 ± 0.96	0.982
N-6/N-3 FA	6.11 ± 1.46^a^	5.27 ± 1.08^a^	5.65 ± 1.54^a^	4.58 ± 1.41^b^	<0.001

^1 ^Probability value obtained by ANOVA. Values followed by the same letter are not significantly different at the 0.05; a, b: p < 0.05 (n 6–10 animals/group).

## Discussion

The three sources of N-3 FA fatty acids were effective in improving the plasma lipid profile of the LDLr knockout mice. Among them, *Echium* oil provided the best results in terms of VLDL and total cholesterol reduction and contributed to the attenuation of hepatic steatosis. None of these oils was able to reduce the inflammation caused by the high fat diet, according to the biomarkers evaluated in this study.

The supplementation with fish and *Echium* oils increased EPA concentrations in liver homogenate. The capacity of SDA to increase EPA content in different tissues is still controversial. Zhang et al. [20] reported that the supplementation of LDLr knockout mice with *Echium* oil (10% of total energy intake) resulted in a significant enrichment of EPA in plasma lipids. According Harris [21] the direct dietary intake of SDA has been proposed to be another strategy to increase tissue EPA levels, since SDA does not depend of Δ6-desaturase to be converted in EPA.

LXRs and PPARs are nuclear receptors that play crucial role in the regulation of fatty acid metabolism [22]. The hypolipidaemic effect of algae and fish oils has been partially attributed to the downregulation of LXRα, with a subsequent inhibition of fatty acid synthesis, associated with the upregulation of PPARα, which promotes β-oxidation. Several studies have demonstrated that EPA and DHA reduce TG and VLDL acting as PPARα agonists and LXRα antagonists [5,23,24]. According to Chilton et al. [3], SDA and EPA reduce the level of mRNA for Sterol Regulatory Element-Binding Protein 1C (SREBP1c), Fatty Acid Synthase (FAS) and stearoyl CoA desaturase 1 (SCD) in liver, suggesting that a possible mechanism to explain TG reduction would be associated with a decrease in the LXRα and consequently in the genes that codify proteins involved in fatty acids hepatic synthesis. This mechanism could be clearly observed to the both marine oils applied in our study, but not to the *Echium* oil. Animals supplemented with *Echium* oil showed the most significant VLDL reduction and attenuated steatosis, although no differences had been in regards to LXRα and PPARα expression. In fact, the mechanisms for the reduction of the plasma TG levels by *Echium* oil are unknown. Although the dose applied in our study was 5-fold lower, our results agree with those reported by Zhang et al. [20], who observed a reduction in TG and VLDL levels after *Echium* oil supplementation without changes in PPARα and LXRα expression. These results suggest that *Echium* oil can exert its beneficial effect on lipid metabolism and hepatic steatosis via mechanisms other than those reported for marine oils. In addition, it has been recommended [25] that studies involving SDA adopt a dose equivalent to EPA for supplementation. However, when this procedure was carried out in our study (Table 3), N-6/N-3 FA ratio of emulsion containing *Echium* oil (6.7) became lower than emulsions with algae (16.7) and fish (16.6) oils. Thus, differences observed in biomarkers between ECH group and the other two supplemented groups (ALG and FIS) can have also been influenced by these difference in N-6/N-3 FA ratio.

Mice supplemented with *Echium* oil showed reduction of N-6/N-3 ratio in liver (Table 5). According to Parker et al. [26] the increase of N-6/N-3 FA ratio in liver is associated with higher steatosis, since this condition can favor lipogenesis and inflammation processes. These findings have also been confirmed in human and animal studies [27,28]. Thus, the lower N-6/N-3 FA ratio in liver homogenate can have contributed to the attenuation of steatosis observed in ECH group.

None of these three N-3 FA fatty acids sources was able to reduce serum inflammatory biomarkers. Ishihara et al. [29] observed that, in whole blood of Balb/c mice, the production of Tumor Necrosis Factor - α (TNFα) was suppressed by ALA, SDA and EPA supplementation. However, the dose applied by the authors was 53-fold higher than the dose used in our study. Our high-fat diet was formulated on basis of the diet applied by Safwat et al. [30] to promote hepatic steatosis in rats. The authors observed that after 10 weeks, the animals developed hepatic steatosis, insulin resistance, hypertriglyceridaemia, and increased VLDL levels, but they observed no evidence of hepatic inflammation or fibrosis, suggesting that the hepatic steatosis was in its early stages. It is possible that the dose of EPA, DHA and SDA used in our study, although corresponding to an intake of 2 g/day for humans, was not sufficient to reduce the inflammation biomarkers when the process is at its initial steps. In spite of this, the high N6/N3 FA ratio present in the diet (16:1), typical of Western diet [5], may have annulled the potential LC N-3 PUFA anti-inflammatory action due to the higher availability of ARA than EPA as substrate to the oxidation mediated by cyclooxygenase and lipooxygenase enzymes.

## Conclusions

In our study, the supplementation with three different sources of N-3 FA fatty acids was evaluated using LDLr knockout animals fed with a high fat diet. It was observed that the best combination of results, in terms of plasma lipid profile and steatosis, was achieved by the supplementation with *Echium* oil, and the mechanism involved in this favourable result seems to be different from those involved with EPA and DHA metabolism, maybe due to the lower N-6/N-3 FA ratio present in the liver of animals supplemented with *Echium* oil. Theoretically, it is possible to transfer a metabolic pathway for EPA and DHA synthesis from a marine organism to an oilseed crop plant [14]. However, while this option is not available, our study confirms that *Echium* oil represents an alternative as natural ingredient to be applied in functional foods to reduce cardiovascular disease risk.

## Abbreviations

ALA: α - Linolenic acid; ALG: Algae oil group; CON: Control group; CRP: C-reactive protein; CVD: Cardiovascular disease; DHA: Docosahexaenoic acid; ECH: Echium oil group; EPA: Eicosapentaenoic acid; FAME: Fatty acid methyl ester; FDA: Food and drug administration; FIS: Fish oil group; HDL: High density lipoprotein; ICAM: Inter-cellular adhesion molecule; IL- 6: Interleukin 6; LDL: Low density lipoprotein; LDL: Receptor deficient mice; LDLr: Knockout mice; LNA: Linoleic acid; LXR- α: Liver X receptor α; LC n-3 PUFA: Long-chain n-3 polyunsaturated fatty acids; N-3 FA: Omega 3 fatty acids; N-6/N-3 FA: Omega 6 fatty acids/omega 3 fatty acids; PPAR-α: Peroxisome proliferator activated receptor α; SDA: Stearidonic acid; TG: Triacylglycerol; TNF- α: Tumor necrosis factor – α; VCAM: Vascular cell adhesion molecule; VLDL: Very low density lipoprotein.

## Competing interest

The authors have no conflict of interest to declare.

## Authors’ contributions

PBB conducted the research and wrote the manuscript. KRM performed part of the experiments. MMR participated in critical revision of the study. IAC contributed to the design of the study, analysis of data, drafting the manuscript and was responsible for the financial support. IAC had primary responsibility for the final content. All the authors read and approved the final manuscript
